# Near-infrared photoimmunotherapy: a comparison of light dosing schedules

**DOI:** 10.18632/oncotarget.17047

**Published:** 2017-04-11

**Authors:** Fusa Ogata, Tadanobu Nagaya, Yuko Nakamura, Kazuhide Sato, Shuhei Okuyama, Yasuhiro Maruoka, Peter L. Choyke, Hisataka Kobayashi

**Affiliations:** ^1^ Molecular Imaging Program, Center for Cancer Research, National Cancer Institute, National Institutes of Health, Bethesda, Maryland, 20892, United States of America

**Keywords:** near infrared photoimmunotherapy, monoclonal antibody, cancer treatment, enhanced permeability and retention effect

## Abstract

Near infrared photoimmunotherapy (NIR-PIT) is a newly-developed cancer therapy in which a monoclonal antibody is conjugated to a near-infrared photoabsorber, IR700 to form an antibody photoabsorber conjugate (APC). After the APC binds to cancer cells expressing the cognate antigen, exposure to NIR light results in rapid, highly selective necrotic cell death of the cancer cells with minimal off-target effects. Several hours after NIR-PIT, the tumor vessels become supraphysiologically permeable and circulating APC can therefore readily leak into the already-treated tumor space where it can bind with viable cancer cells that is called super-enhanced permeability and retention effect. The presence of the SUPR effect after NIR-PIT has prompted regimens in which there is a repeat exposure of NIR light 24 hours after the initial NIR-PIT to take advantage of the leakage of additional APC deeper into the tumor. However, this post-treatment APC penetration was fully induced within 3 hours, therefore, it is possible that repeated exposures of NIR light could be administered much earlier than 24 hours and still produce the same effects. To test this idea, we compared several modes of delivering additional doses of light after initial NIR-PIT. We found that repeated exposures of NIR light starting 3 hours after initial NIR-PIT produced equal or superior results to more delayed exposures of NIR light. This finding has practical implications of an easy-to-perform regimen as repeated light exposures could be performed during a single day rather than extending the procedure over two days which is the current recommendation.

## INTRODUCTION

Traditional methods of treating cancers include surgery, radiation and chemotherapy each of which is associated with well-known morbidities. Antibody-based drugs were designed to target specific cancer cells by binding to cell surface antigens with high specificity and affinity [1–3]. Unfortunately, monoclonal antibodies (mAb) by themselves are not usually effective against solid tumors. However, the concept of arming antibodies with drugs, radioisotopes or other mechanisms have been proposed and have resulted in several successful therapies. We developed a new cancer therapy, termed near infrared photoimmunotherapy (NIR-PIT) that relies on the conjugation of a photoabsorber to the antibody. Exposure to NIR light selectively induces necrotic cell death only in target-expressing cancer cells, thus, minimizing the risk of serious side effects [4]. NIR-PIT employs a targeted mAb conjugated to the photoabsorber, IRDye700DX (IR700, silica-phthalocyanine dye) to form an antibody photoabsorber conjugate (APC) [4]. When an APC is administered to a patient and binds a tumor that expresses the cognate antigen, subsequent exposure to NIR light (690 nm), induces rapid and irreversible damage to the cell membrane leading to necrotic/immunogenic cell death [5]. Within minutes of NIR light exposure, target-expressing cells become swollen and burst, even while adjacent non-targeted expressing cells are completely undamaged [6–8]. Recently, new type of cancer photodynamic therapies using genetically transfected photo-toxic fluorescent proteins were reported. Cancer cells expressing such fluorescent proteins were successfully treated with exposure of ultraviolet C [9, 10]. The agents in the nucleus and/or the cytoplasm react with the light and results in the apoptotic cell death due to oxidation reaction. In contrast, NIR-PIT agents on the cell membrane react with the light and result rapid necrotic cell death due to photo-chemical reaction [7, 9, 10].

A Phase I/II trial of NIR-PIT in patients with recurrent head and neck cancer (ClinicalTrials.gov Identifier: NCT02422979) is underway. In this trial, cetuximab is conjugated to IR700 (RM-1929) and this APC is injected systemically. Twenty four hours after administration of RM-1929, the patient receives NIR light via laser. Subsequent re-exposure to the drug and NIR light are allowed in this trial if residual disease is found. The trial has recently advanced to Phase II. Preliminary results suggest NIR-PIT is safe and effective (personal communication). However, the course of therapy, by design, takes place over many weeks.

An interesting aspect of NIR-PIT is that the vasculature becomes very leaky for several hours after treatment. Since the APC initially leaks into the perivascular tissues of the tumor, NIR-PIT tends to preferentially kill tumor cells adjacent to vessels, at least initially. This results in dramatic, if short-lived, increases in vascular permeability as the cells crowding the vessel are no longer present. This effect is termed super enhanced permeability and retention (SUPR) to differentiate it from the commonly observed enhanced permeability and retention (EPR) which is a common property of untreated tumor vasculature. Studies have shown that NIR-PIT can result in substantial (up to 24-fold) increases in the leakage of nano-sized drugs and APCs into treated tumor beds [11]. The SUPR effect opens up possibilities for adjuvant nanotherapies following NIR-PIT. Because the initial APC is photo-bleached by exposure to NIR light, fresh circulating APC is needed if additional NIR-PIT is to be performed. SUPR provides a mechanism by which the tumor can be refreshed with unexposed APC, thus enabling additional NIR-PIT treatments [12, 13–16].

Repeated doses of NIR light at 24 hours after initial NIR-PIT is known to improve outcomes in pre-clinical models. However, the APC refreshment within the tumor after initial NIR-PIT was fully induced within 3 hours, so that it is possible that repeat light exposures, including more than just one, might be possible much earlier than 24 hours. Therefore, the purpose of this study was to compare the efficacy of multiple NIR light exposures at different light doses and schedules after initial NIR-PIT.

## RESULTS

### Repeated NIR-PIT increases pan-IR700 delivery

**Figure 1:**
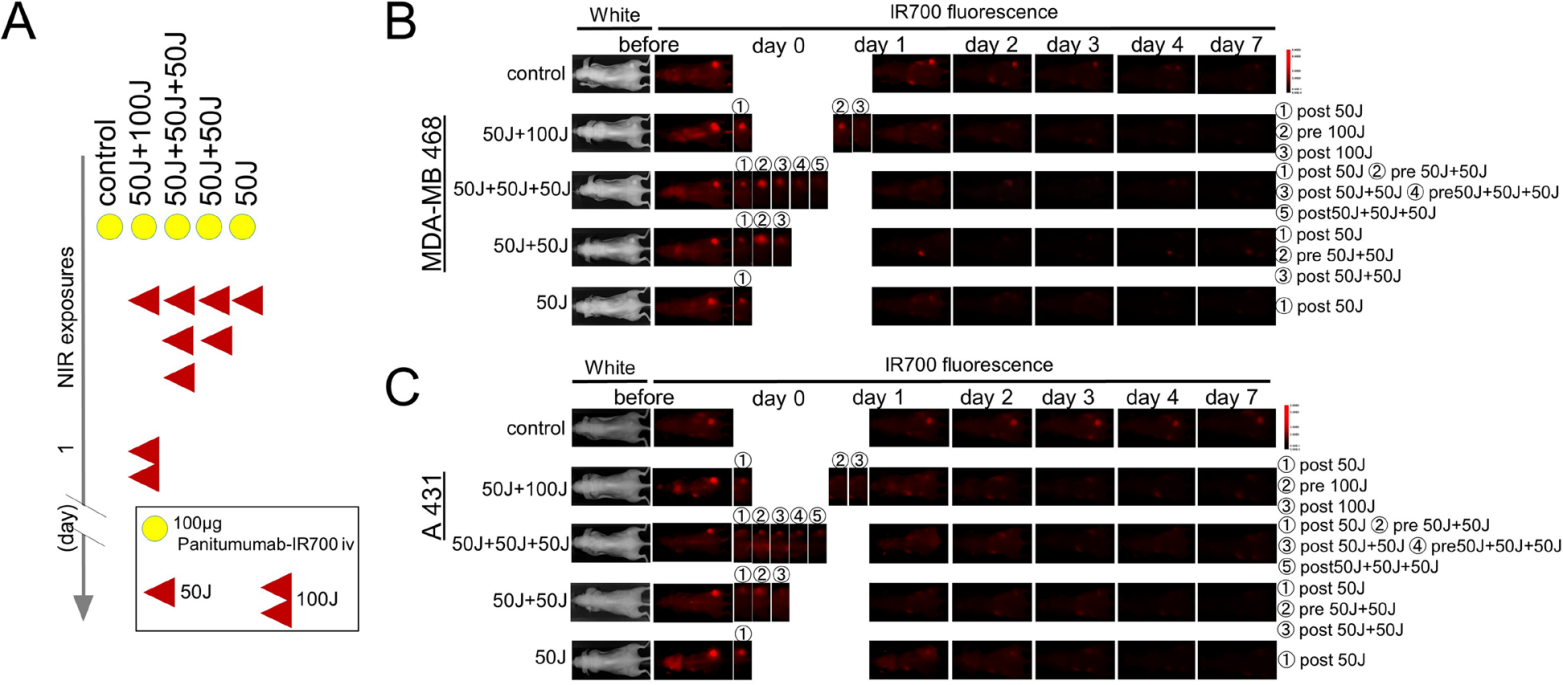
IR700 fluorescence real-time imaging in MDA-MB468luc tumors and A431luc tumors (**A**) Treatment schedule demonstrating the regimen of NIR-PIT with multiple NIR light exposures. (**B, C**) IR700 fluorescence real-time imaging in MDA-MB468luc tumors (B) and A431luc tumors (C). The distribution of Pan-IR700 is demonstrated.

**Figure 2:**
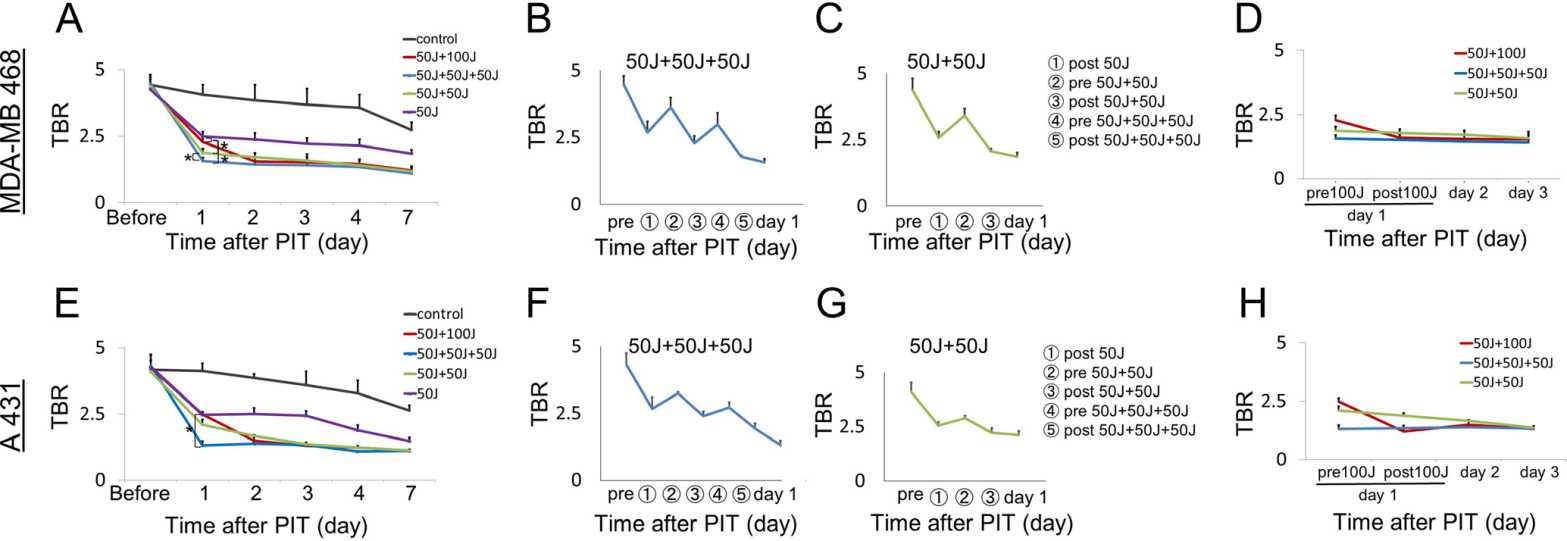
Quantitative analysis of IR700 fluorescence intensities in MDA-MB468luc tumors (A–D) and A431luc tumors (E–H) (**A**, **D**, **E**, **H**) The fluorescence intensity on day 1 significantly decreased in proportion to the total light dose in treated groups due to photobleaching but then increased again due to enhanced leakage of non-irradiated circulating APC into the tumor. However, there were no significant differences in the intensity on day 2 among the groups receiving multiple NIR light exposures (50 J/cm^2^ + 50 J/cm^2^, 50 J/cm^2^ + 50 J/cm^2^ + 50 J/cm^2^, 50 J/cm^2^ + 100 J/cm^2^). The fluorescence intensity gradually decreased at seven days after the last NIR-PIT due to excretion of the APC. (**B**, **C**, **F**, **G**) The fluorescence intensity after each NIR light exposure dramatically changed with intensities decreasing after every NIR light exposure. Data are means ± SEM. *n* = 5 in each group. *P < 0.05 versus the other group.

Since IR700 is a fluorescent dye, Pan-IR700 is fluorescent at lower levels of excitation NIR light [4, 8]. However, therapeutic light levels will result in photobleaching and decreased fluorescence. To quantitatively evaluate the fluorescence intensity of Pan-IR700 before and after irradiation in both tumor types, the target-to-background ratio (TBR) was assessed. IR700 fluorescence intensity in controls decreased slowly with time commensurate the conjugate's pharmacokinetics and washout of the APC from the tumor. In contrast, the TBR in treated groups markedly decreased immediately after NIR irradiation due to photobleaching and then continued to decrease over the following days due to washout (Figure 1B, 1C). The fluorescence intensities on day 1 (before second irradiation of 50 or 100 J/cm^2^) significantly decreased in proportion to the dose of light and the number of prior irradiations. In the group given NIR 24 hours after the first irradiation (50 J/cm^2^ + 100 J/cm^2^), for a total dose of 150 J/cm^2^, the serial TBR after day 2 was not significantly different from the group that received an additional 50 J/cm^2^ at 24 hours for a total dose of 100 J/cm^2^. Moreover, the intensities on day 2 in the groups receiving multiple NIR light exposures (50 J/cm^2^ + 50 J/cm^2^ in same day, 50 J/cm^2^ + 50 J/cm^2^ + 50 J/cm^2^ in same day, 50 J/cm^2^ + 100 J/cm^2^ in different days) were not significantly different (Figure 2D, 2H) and after that they gradually decreased over the seven days they were observed (Figure 2A, 2E). As previously reported, NIR-PIT increased leakage of pan-IR700, into treated tumors up to 6 to 8 hours after irradiation [14]. In the groups receiving multiple light exposures in same day (50 J/cm^2^ + 50 J/cm^2^, 50 J/cm^2^ + 50 J/cm^2^ + 50 J/cm^2^), the fluorescence intensities initially dropped due to photobleaching but then almost immediately recovered as fresh APC entered the tumor bed before the next NIR light exposure whereupon the process was repeated. After every light exposure there was a sufficient decrease in TBR likely resulting from the combination of permanent photobleaching and excretion of the APC (Figure 2B, 2C, 2F, 2G). The recovery of the fluorescence after NIR light exposure relies on the SUPR effect, enabling repeated light exposures on the same day. Since NIR-PIT can specifically kill target cancer cells without damaging normal cells [4] including vascular endothelial cells, stromal cells and immune cells [17], the circulating pan-IR700 can leak into tumor beds and bind to the remaining cancer cells via the permeable endothelium of undamaged blood vessels. These results suggest that repeated NIR light exposures, within a few hours of initial NIR-PIT, can enhance therapeutic efficacy even with lower doses of light.

### Repeated NIR light exposure enhances tumor killing

**Figure 3:**
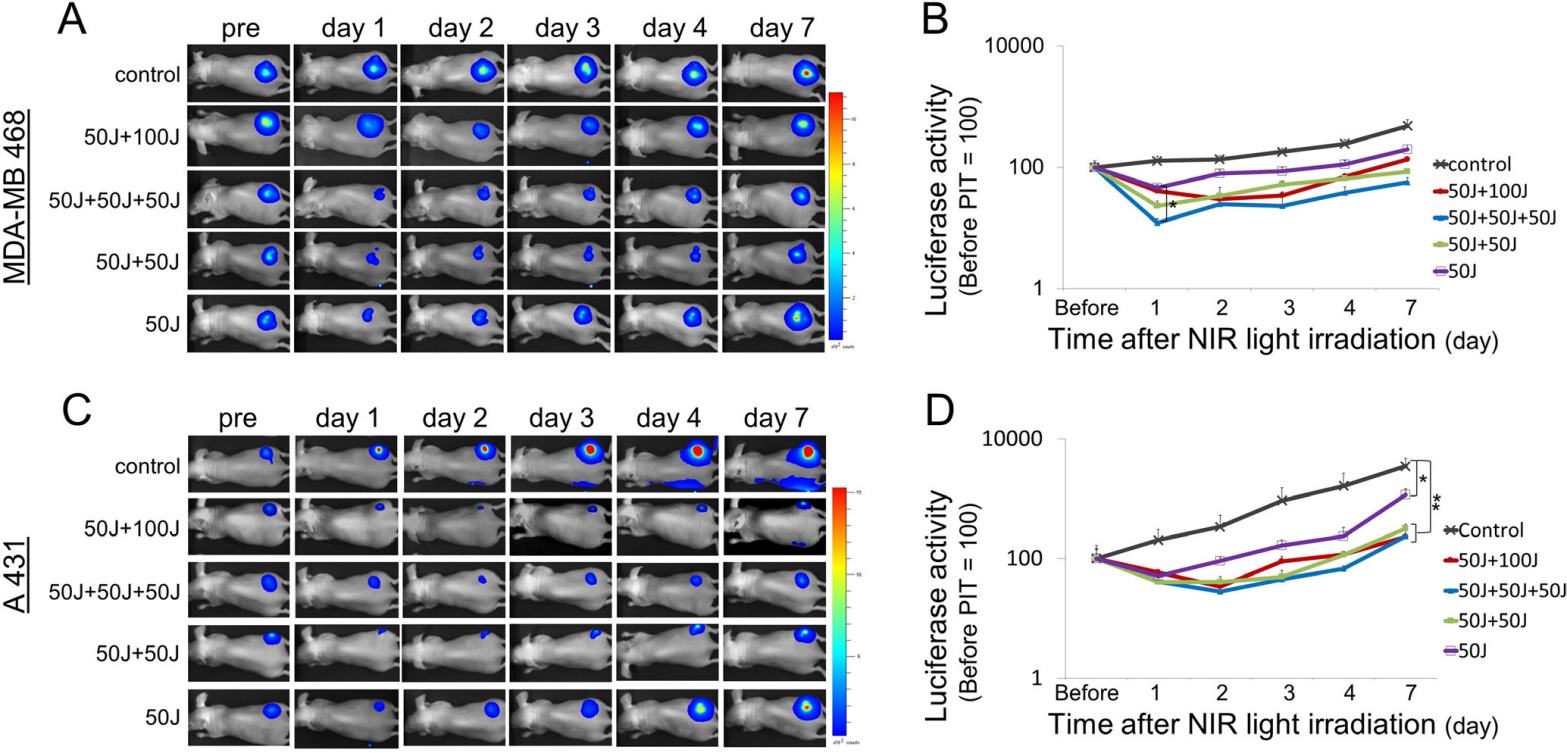
Bioluminescence imaging in MDA-MB468luc tumors and A431luc tumors (**A**, **C**) In all the treated groups the signal significantly decreased one day after each NIR light exposure and then gradually increased due to tumor regrowth. (**B**, **D**) Quantitative analysis of bioluminescence signals in MDA-MB468luc tumors (B) and A431luc tumors (D). Data are means ± SEM. *n* = 5 in each. *P < 0.05, **P < 0.005 versus the other group. Although there was a significant difference only between 50 J/cm^2^ and 50 J/cm^2^ + 50 J/cm^2^+ 50 J/cm^2^ on day 1 (B), the signal in the groups with repeated NIR light exposures showed a tendency to decrease as a function of light dose.

In parallel with IR700 fluorescence imaging, we also performed bioluminescence imaging (BLI) before and after NIR light exposure up to day 7 in MDA-MB468luc tumors and A431luc tumors. BLI is a highly sensitive tool for evaluating live cells after NIR-PIT as it depends on the catalysis of luciferin by luciferase mediated by oxygen and ATP [18, 19]. In all treated groups the BLI signal decreased immediately after NIR-PIT and then gradually increased (Figure 3A, 3C). This pattern of massive loss of BLI followed by slow recovery is likely due to a large amount of initial cell killing followed by slower regrowth of cells not originally killed. Comparing groups, the greatest reduction in BLI signal occurred in the group that received 3 light doses (50 J/cm^2^ + 50 J/cm^2^ + 50 J/cm^2^) on day 1. Although the results were not statistically significant for the treated groups on day 1 (*P >* 0.05, Tukey-Kramer), the BLI was most reduced in the groups receiving multiple light doses (Figure 3B, 3D). However, by day 7, BLI was significantly lower in the groups undergoing repeated light doses in the first day in A431 tumor (*P* < 0.005, Tukey-Kramer). These results suggest that multiple light exposures during the first day are at least comparable, if not superior to, light exposure on sequential days.

### Repeated NIR-PIT prolongs overall survival

**Figure 4:**
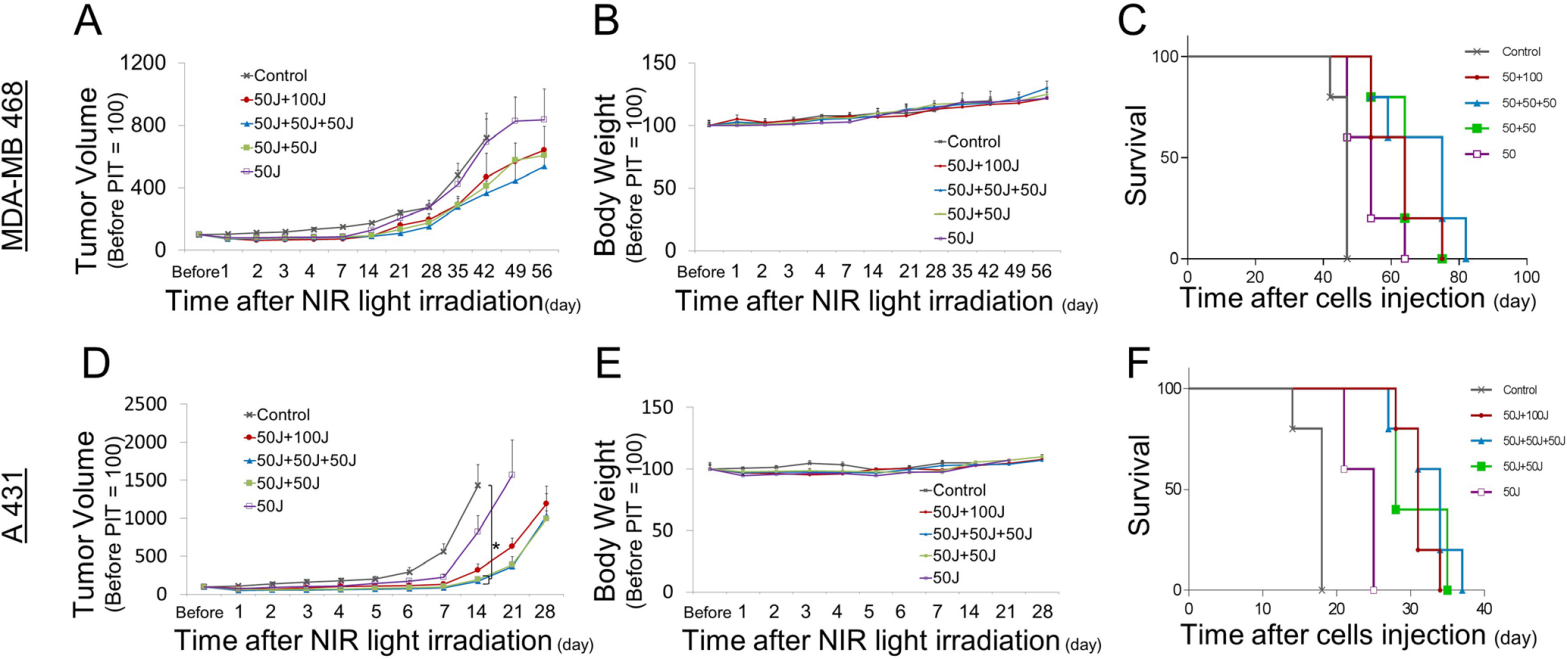
Long-term observations after NIR-PIT (**A**, **D**) Tumor growth was significantly reduced by repeated exposures to NIR light. (**B**, **E**) Body weights showed no significant difference among groups. (**C**, **F**) Kaplan-Meier survival curve of mice treated with NIR-PIT.

An additional outcome measure in this study was overall survival. Survival in the context of rodent studies often is a surrogate for tumor size as animal care and use guidelines prescribe euthanasia of animals when their tumor reaches a certain size (2 cm in our center), in the absence of other signs of distress. Thus, external caliper measurements of the tumor were also used. The multiple NIR light exposure groups showed the slowest rate of tumor regrowth compared with the single irradiation group (Figure 4A, 4D). Despite the lower total light dose, NIR light exposure repeated twice within three hours (50 J/cm^2^ + 50 J/cm^2^) induced equivalent therapeutic effects with the other multi light exposure regimens; (50 J/cm^2^ + 50 J/cm^2^ + 50J /cm^2^, 50 J/cm^2^ + 100 J/cm^2^) (Figure 4A, 4D). Survival was significantly prolonged in the groups receiving multiple light exposure vs. those that had received only a single light exposure (Figure 4C, 4F). There were no significant differences in body weight among the mice given the various light dosing regimens, suggesting minimal acute toxicity due to NIR-PIT (Figure 4B, 4E). Thus, multiple light exposures after the initial administration of APC and NIR light exposure inhibits tumor regrowth leading to prolonged overall survival without any additional adverse side-effects.

## DISCUSSION

NIR-PIT is a highly selective and effective cancer treatment for tumors in which NIR light can be readily delivered to the tumor [17, 20–24]. The preferred regimen has been to inject an APC approximately 24 hours prior to initial NIR light exposure. A second light exposure 24 hours later has been suggested to allow circulating APC to penetrate into the remaining tumor and then be activated by as subsequent NIR light exposure about 24 hours later. However, NIR-PIT immediately induces a profound increase in vascular leakiness, known as SUPR, for approximately 6–8 hours. Therefore, we reasoned that additional NIR light doses might be repeated earlier than 24 hours and achieve equal or superior results. Multiple doses of light on the same day might make NIR-PIT more efficient as a patient would not have to return to the treatment center on another day. We compared four NIR light dosing regimens in MDA-MB468luc tumors and A431luc tumor models including a single irradiation (50 J/cm^2^, total dose 50 J/cm^2^), 3 repeats of NIR light every three hours (50 J/cm^2^ + 50 J/cm^2^ + 50 J/cm^2^, total dose 150 J/cm^2^), 2 repeats of NIR light at three hour intervals after the first irradiation (50 J/cm^2^ + 50 J/cm^2^, total dose 100 J/cm^2^) and a repeat NIR light exposure 24 hours later (50 J/cm^2^ + 100 J/cm^2^, total 150 J/cm^2^). The last regimen was assumed to be the “standard” protocol to be used as it was the one originally tested. The groups that received 2 or 3 NIR light exposures demonstrated similar effectiveness as measured by BLI and tumor volume. Since MDA-MB468luc tumors grew slower than A431luc tumors mice with MDA-MB468luc tumors survived longer than those with A431 tumors. However, therapeutic effects of NIR-PIT of different regimens were identical in both tumor models.

A clinical trial of NIR-PIT is underway in head and neck cancer patients. In this trial (NCT02422979) a single dose of cetuximab-IR700 is administered and the patient receives NIR light the following day. If there is residual disease the patient is retreated with a new administration of drug and light several weeks later. The results of the current study could potentially pave the way for a more convenient regimen in which a single dose of the drug is followed by multiple exposures to NIR light on the same day. Shortening the treatment time and number of anesthesia exposures could substantially reduce the cost of the procedure and as well as yield more immediate benefits [25, 26]. These benefits need to be balanced against longer anesthesia exposures required for the multi light dose regimen performed on a single day.

## MATERIALS AND METHODS

### Cells

HER1-expressing A431luc cells, a human epidermoid carcinoma cell line, and HER1-expressing MDA-MB468luc cells, a human breast cancer cell line, were grown in RPMI 1640 supplemented with 10% fetal bovine serum and 1% penicillin/streptomycin in tissue culture flasks in a humidified incubator at 37°C in an atmosphere of 95% air and 5% carbon dioxide.

### Synthesis of panitumumab-IR700 conjugates (pan-IR700)

Panitumumab (1 mg, 6.8 nmol) was incubated with IR700 (66.8 μg, 34.2 nmol, 5 mmol/L in DMSO) in 0.1 mol/L Na_2_HPO_4_ (pH 8.5) at room temperature for 2 h. The mixture was purified with a Sephadex G25 column (PD-10; GE Healthcare, Waukesha, WI). The protein concentration was determined with Coomassie Plus protein assay kit (Thermo Fisher Scientific Inc., Rockford, IL) by measuring the absorption at 595 nm with spectroscopy (8453 Value System; Agilent Technologies, Santa Clara, CA). The concentration of IR700 was measured by absorption with spectroscopy to confirm the number of fluorophore molecules conjugated to each mAb molecule.

### Murine tumor models

All *in vivo* procedures were conducted in compliance with the Guide for the Care and Use of Laboratory Animal Resources (1996), US National Research Council, and approved by the local Animal Care and Use Committee. 6- to 8-week-old female homozygote athymic nude mice were purchased from Charles River (NCI, Frederick, MD). During procedures, mice were anesthetized with isoflurane (5% for induction; 2% for maintenance). Tumor models were established by injecting four million A431luc cells or eight million MDA-MB468luc cells subcutaneously in the right dorsum of the mice. The tumor volume was based on caliper measurements, the greatest longitudinal diameter (length) and the greatest transverse diameter (width), were used to calculate tumor volume = length × width^2^ × 0.5 [27].

### NIR-PIT for MDA-MB468 cells or A431 cells with pan-IR700

Tumors reaching approximately 50 mm^3^ in volume were selected for further experiments and the mice were divided randomly into five experimental groups of 5 mice per group for the following treatments. In all groups 100 μg of pan-IR700 was administered intravenously one day before NIR light exposure: (1) no NIR light irradiation (control) (2) NIR light administered at 50 J/cm^2^ on day 0 and 100 J/cm^3^ on day 1 (the standard protocol) (3) NIR light was administered at 50 J/cm^3^ three times at three hour intervals on day 0 (4) NIR light administered at 50 J/cm^3^ twice at three hour intervals on day 0 (5) NIR light was administered once at 50 J/cm^3^ on day 0. Pan-IR700 stably accumulates in treated tumors 24 hours after administration [28] and thereby we applied the first NIR light exposure on day 1.

### Evaluation of the SUPR effect with pan-IR700

Pan-IR700 is normally fluorescent with low levels of excitation light. Exposure of Pan-IR700 to higher doses of NIR results in bleaching. Therefore, the presence of fresh unexposed APC within the tumor can be inferred by a gain in fluorescence after NIR-PIT. Serial fluorescence images were assessed before and after a NIR light exposure (Figure 1A) using a Pearl Imager (LI-COR Biosciences, Lincoln, NE, USA) with a 700 nm fluorescence channel. For analyzing fluorescence intensities, mean intensities of IR700 signal in each the region of interest were calculated with Pearl Cam Software (LICOR Biosciences). A region of interest (ROI) was placed on the tumor and the average fluorescence intensity of IR700-signal was calculated for each ROI. The TBR was calculated from fluorescence intensities of tumor and fluorescence intensities of background.

### Bioluminescence imaging studies after NIR-PIT

Bioluminescence can be used to determine cell viability since it depends on the presence of actively generated cellular ATP. D-luciferin (15 mg/mL, 200 μL, Gold Biotechnology, St. Louis, MO) was injected intraperitoneally into mice and analyzed with Photon Imager (LI-COR Biosciences, Lincoln, NE, USA) for luciferase activity indicative of tumor cell viability. Serial images were assessed before and after NIR light exposure day 0 and day 1 (Figure 1A).

### Statistical analysis

Comparisons between groups were made using Student's *t* test (2-tailed). Differences among more than two groups were analyzed using one-way analysis of variance (ANOVA) followed by post hoc Bonferroni (3 groups) or Tukey-Kramer (> 3 groups) tests. Values of *P* less than 0.05 were considered significant. Error bars represent standard error of the mean (SEM). The cumulative probability of survival was estimated in each group with the use of the Kaplan–Meier survival curve analysis, and the results were compared with use of the log-rank test with Bonferroni's correction for multiplicity. However, tumor bearing mice were euthanized for ethical reasons when tumor size reached 20 mm in length or there were signs of distress owing to tumor growth or ulceration.

## CONCLUSIONS

One day after receiving a single dose of an APC, repeated NIR light exposures on the same day at three hour-intervals yielded similar results to existing 2-day regimens and superior results to single light exposure regimens. This suggests a potentially easy-to-perform method of improving the efficiency of NIR-PIT without fundamental changes in the manner in which it is currently performed.

## Abbreviations

ANOVA: one-way analysis of variance
APC: antibody-photoabsorber conjugate
ATP: adenosine triphosphate
BLI: bioluminescence imaging
DMSO: Dimethyl sulfoxide
EPR: enhanced permeability and retention
HER1: Epidermal Growth Factor Receptor
IR700: IRDye700DX
mAb: monoclonal antibodies
NIR: near-infrared
mAb: monoclonal antibody
PIT: photoimmunotherapy
ROI: region of interest
SUPR: super enhanced permeability and retention
TBR: target-to-background ratio

## References

[1] Waldmann TA (2003). Immunotherapy: past, present and future. Nat Med.

[2] Wu AM, Senter PD (2005). Arming antibodies: prospects and challenges for immunoconjugates. Nat Biotechnol.

[3] Couzin-Frankel J (2013). Breakthrough of the year 2013. Cancer immunotherapy. Science.

[4] Mitsunaga M, Ogawa M, Kosaka N, Rosenblum LT, Choyke PL, Kobayashi H (2011). Cancer cell-selective *in vivo* near infrared photoimmunotherapy targeting specific membrane molecules. Nature medicine.

[5] Ogawa M, Tomita Y, Nakamura Y, Lee MJ, Lee S, Tomita S, Nagaya T, Sato K, Yamauchi T, Iwai H, Kumar A, Haystead T, Shroff H (2017). Immunogenic cancer cell death selectively induced by near infrared photoimmunotherapy initiates host tumor immunity. Oncotarget.

[6] Sano K, Mitsunaga M, Nakajima T, Choyke PL, Kobayashi H (2013). Acute Cytotoxic Effects of Photoimmunotherapy Assessed by 18F-FDG PET. Journal of nuclear medicine.

[7] Mitsunaga M, Nakajima T, Sano K, Kramer-Marek G, Choyke PL, Kobayashi H (2012). Immediate *in vivo* target-specific cancer cell death after near infrared photoimmunotherapy. BMC cancer.

[8] Nakajima T, Sano K, Mitsunaga M, Choyke PL, Kobayashi H (2012). Real-time monitoring of *in vivo* acute necrotic cancer cell death induced by near infrared photoimmunotherapy using fluorescence lifetime imaging. Cancer research.

[9] Kimura H, Lee C, Hayashi K, Yamauchi K, Yamamoto N, Tsuchiya H, Tomita K, Bouvet M, Hoffman RM (2010). UV light killing efficacy of fluorescent protein-expressing cancer cells *in vitro* and *in vivo*. J Cell Biochem.

[10] Momiyama M, Suetsugu A, Kimura H, Kishimoto H, Aki R, Yamada A, Sakurada H, Chishima T, Bouvet M, Bulgakova NN, Endo I, Hoffman RM (2012). Fluorescent proteins enhance UVC PDT of cancer cells. Anticancer Res.

[11] Maeda H (2010). Tumor-selective delivery of macromolecular drugs via the EPR effect: background and future prospects. Bioconjug Chem.

[12] Liang CP, Nakajima T, Watanabe R, Sato K, Choyke PL, Chen Y, Kobayashi H (2014). Real-time monitoring of hemodynamic changes in tumor vessels during photoimmunotherapy using optical coherence tomography. Journal of biomedical optics.

[13] Mitsunaga M, Nakajima T, Sano K, Choyke PL, Kobayashi H (2012). Near-infrared theranostic photoimmunotherapy (PIT): repeated exposure of light enhances the effect of immunoconjugate. Bioconjugate chemistry.

[14] Sano K, Nakajima T, Choyke PL, Kobayashi H (2013). Markedly enhanced permeability and retention effects induced by photo-immunotherapy of tumors. ACS Nano.

[15] Sano K, Nakajima T, Choyke PL, Kobayashi H (2014). The effect of photoimmunotherapy followed by liposomal daunorubicin in a mixed tumor model: a demonstration of the super-enhanced permeability and retention effect after photoimmunotherapy. Molecular cancer therapeutics.

[16] Hanaoka H, Nakajima T, Sato K, Watanabe R, Phung Y, Gao W, Harada T, Kim I, Paik CH, Choyke PL, Ho M, Kobayashi H (2015). Photoimmunotherapy of hepatocellular carcinoma-targeting Glypican-3 combined with nanosized albumin-bound paclitaxel. Nanomedicine (Lond).

[17] Sato K, Sato N, Xu B, Nakamura Y, Nagaya T, Choyke PL, Hasegawa Y, Kobayashi H (2016). Spatially selective depletion of tumor-associated regulatory T cells with near-infrared photoimmunotherapy. Science translational medicine.

[18] Wilson T, Hastings JW (1998). Bioluminescence. Annu Rev Cell Dev Biol.

[19] Contag CH, Bachmann MH (2002). Advances in *in vivo* bioluminescence imaging of gene expression. Annu Rev Biomed Eng.

[20] Sato K, Hanaoka H, Watanabe R, Nakajima T, Choyke PL, Kobayashi H (2015). Near infrared photoimmunotherapy in the treatment of disseminated peritoneal ovarian cancer. Molecular cancer therapeutics.

[21] Sato K, Nagaya T, Choyke PL, Kobayashi H (2015). Near infrared photoimmunotherapy in the treatment of pleural disseminated NSCLC: preclinical experience. Theranostics.

[22] Nagaya T, Nakamura Y, Sato K, Harada T, Choyke PL, Hodge JW, Schlom J, Kobayashi H (2017). Near infrared photoimmunotherapy with avelumab, an anti-programmed death-ligand 1 (PD-L1) antibody. Oncotarget.

[23] Nagaya T, Nakamura Y, Sato K, Zhang YF, Ni M, Choyke PL, Ho M, Kobayashi H (2016). Near infrared photoimmunotherapy with an anti-mesothelin antibody. Oncotarget.

[24] Nakamura Y, Bernardo M, Nagaya T, Sato K, Harada T, Choyke PL, Kobayashi H (2016). MR imaging biomarkers for evaluating therapeutic effects shortly after near infrared photoimmunotherapy. Oncotarget.

[25] Hutchinson L, Kirk R (2011). High drug attrition rates--where are we going wrong?. Nat Rev Clin Oncol.

[26] Yabroff KR, Lund J, Kepka D, Mariotto A (2011). Economic burden of cancer in the United States: estimates, projections, and future research. Cancer Epidemiol Biomarkers Prev.

[27] Euhus DM, Hudd C, LaRegina MC, Johnson FE (1986). Tumor measurement in the nude mouse. J Surg Oncol.

[28] Nagaya T, Sato K, Harada T, Nakamura Y, Choyke PL, Kobayashi H (2015). Near Infrared Photoimmunotherapy Targeting EGFR Positive Triple Negative Breast Cancer: Optimizing the Conjugate-Light Regimen. PloS one.

